# Thermal Image Scanning for Influenza Border Screening: Results of an Airport Screening Study

**DOI:** 10.1371/journal.pone.0014490

**Published:** 2011-01-05

**Authors:** Patricia C. Priest, Alasdair R. Duncan, Lance C. Jennings, Michael G. Baker

**Affiliations:** 1 Department of Preventive and Social Medicine, University of Otago, Dunedin, New Zealand; 2 Planning and Funding, Canterbury District Health Board, Christchurch, New Zealand; 3 Virology Section, Canterbury Health Laboratories, Christchurch, New Zealand; 4 Department of Public Health, University of Otago Wellington, Wellington, New Zealand; The University of Hong Kong, Hong Kong

## Abstract

**Background:**

Infrared thermal image scanners (ITIS) appear an attractive option for the mass screening of travellers for influenza, but there are no published data on their performance in airports.

**Methods:**

ITIS was used to measure cutaneous temperature in 1275 airline travellers who had agreed to tympanic temperature measurement and respiratory sampling. The prediction by ITIS of tympanic temperature (37.8°C and 37.5°C) and of influenza infection was assessed using Receiver Operating Characteristic (ROC) curves and estimated sensitivity, specificity and positive predictive value (PPV).

**Findings:**

Using front of face ITIS for prediction of tympanic temperature ≥37.8°C, the area under the ROC curve was 0.86 (95%CI 0.75–0.97) and setting sensitivity at 86% gave specificity of 71%. The PPV in this population of travellers, of whom 0.5% were febrile using this definition, was 1.5%. We identified influenza virus infection in 30 travellers (3 Type A and 27 Type B). For ITIS prediction of influenza infection the area under the ROC curve was 0.66 (0.56–0.75), a sensitivity of 87% gave specificity of 39%, and PPV of 2.8%. None of the 30 influenza-positive travellers had tympanic temperature ≥37.8°C at screening (95%CI 0% to 12%); three had no influenza symptoms.

**Conclusion:**

ITIS performed moderately well in detecting fever but in this study, during a seasonal epidemic of predominantly influenza type B, the proportion of influenza-infected travellers who were febrile was low and ITIS were not much better than chance at identifying travellers likely to be influenza-infected. Although febrile illness is more common in influenza A infections than influenza B infections, many influenza A infections are afebrile. Our findings therefore suggest that ITIS is unlikely to be effective for entry screening of travellers to detect influenza infection with the intention of preventing entry of the virus into a country.

## Introduction

Rising concerns regarding Influenza A (H5N1) and the pandemic of Influenza A (H1N1) 2009 have led to the use of infrared thermal image scanners (ITIS) at some borders for the mass screening of travellers to detect those who might be infected with influenza [1]. ITIS measure body surface temperature rapidly, non-invasively, and with no contact, minimising the risk of contagion. They therefore have the potential to comply with the International Health Regulations' emphasis on containing the spread of disease in ways that avoid unnecessary interference with international traffic and trade [2].

Evaluations of the use of ITIS in clinical settings have been conducted, and have reported sensitivities of 15% to 90% for confirmed fever depending on the cut-off used to define fever [3], [4], [5], [6]. However, these findings may not be applicable to border screening. ITIS measure body surface temperature, not body core temperature, and so ITIS temperature measurements are subject to the influence of a range of human and environmental factors. These include whether a person is sunburnt, has taken antipyretics or has circulatory problems, and also the ambient temperature and humidity. Consequently it is important that the relationship between body surface temperature and body core temperature be evaluated within the environment in which ITIS are to be operated.

In the airport setting, thermal scanning of arriving travellers has been used to screen for several different infectious diseases. During the outbreak of Severe Acute Respiratory Syndrome (SARS) ITIS use was documented, however only the numbers of travellers triggering the scanner were reported, without stating the cut-off threshold used for fever or reporting on any subsequent method used to confirm febrile status [7], [8], [9]. A trial dengue fever screening programme found that among travellers arriving into Cairns airport [10] 12% (118/963) of travellers who triggered the pre-set alarm threshold were confirmed to be febrile on tympanic temperature measurement. Influenza screening in Singapore found that only 12% of cases of pandemic (H1N1) 2009 infection with onset within 10 days of arrival were detected by ITIS on entry [11].

Proper evaluation of a screening test requires that the ‘gold standard’ test is applied to both test positive and test negative participants in the study. To evaluate the use of ITIS in border screening for influenza, its performance in predicting both fever and also influenza infection is necessary. However to date no studies have been reported that tested ITIS negative travellers for either fever or influenza infection [12], [13].

We undertook both ITIS and tympanic temperature measurement on, and collected specimens for testing for influenza from, symptomatic and asymptomatic air travellers arriving into Christchurch, New Zealand during the Southern hemisphere winter in 2008. This paper assesses the performance of ITIS in detection of fever and infection with seasonal influenza in these airline travellers.

## Methods

### Study design

This evaluation of thermal image scanning was carried out as part of a larger study to measure the prevalence of seasonal influenza infection in arriving airline travellers and the effectiveness of a screening questionnaire for detecting those with influenza infection. The design followed closely a pilot study carried out in 2007 [14].

### Participants

Three airlines agreed to have their staff distribute a screening questionnaire to travellers (passengers and crew) during flights travelling from Australian airports to Christchurch, New Zealand. The questionnaires were collected by research assistants following immigration processing on arrival in Christchurch. ‘Symptomatic’ travellers were defined as those who reported one or more of the following symptoms: cough, sore throat, sneezing, fever or chills, runny or blocked nose, muscle aches or pains, feeling generally unwell, chest discomfort or breathing difficulties.

### Measures

Symptomatic travellers were all invited to have throat and nose swabs (Copan Italia SPA, Brescia, Italy) taken and their temperature measured. In addition, half the questionnaires were marked and were randomly placed into the sets of questionnaires delivered to the flight crew (the sequence was determined by the RAND function of Microsoft Excel©). Arriving travellers carrying a marked questionnaire were also invited to have swabs and temperature taken. The nurse taking the swabs noted on the request form whether the traveller was symptomatic or asymptomatic.

**Figure 1:**
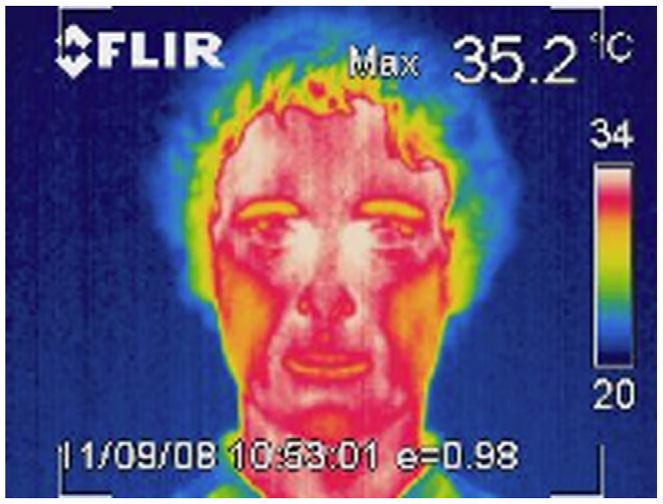
ITIS image of front of face.

For the 23 working days from 21 August to 12 September 2008, cutaneous temperature from those travellers invited to participate who had given consent was measured using ITIS (ThermaCAM™ E45, FLIR Systems, Sweden) prior to swabs being taken. A focal plane array (160×120 pixels) was used on the front of the face and the side of the face (see Figure 1) and the maximum temperature reading for each was recorded. After the swabs were taken, each participant's tympanic temperature was measured using an infrared tympanic thermometer (ThermaScan PRO4000, BRAUN, Germany).

The ambient temperature in the arrivals hall was a consistent 20.5°C at all times during data collection.

### Laboratory Analysis

All nasal and throat swab samples were analysed at Canterbury Health Laboratories, Christchurch. A multiplexed tandem polymerase chain reaction (MT-PCR) assay was employed to detect the presence of influenza A and B virus infection, as described by the manufacturer (Easy-Plex Influenza A+B kit, Cat. No. 3005.01, AusDiagnostics Pty Ltd, Sydney, Australia).

### Data Analysis

Stata© 10 was used to analyse the data. The cii command was used to calculate Poisson exact confidence intervals around the proportion of influenza-infected travellers who were febrile.

Information about temperature measurements was collected on the swab consent form and linked to the symptom information on the questionnaire using a unique swab identifier. Nine swab results were unable to be linked as their identifier had not been attached to any questionnaire. For these individuals the nurse's note of whether or not they were symptomatic was used to define their symptom status.

Analyses were performed to assess the accuracy of ITIS measurements in predicting two different tympanic temperature thresholds:

1. tympanic temperature ≥37.8°C (>100°F – the level used by the Centers for Disease Control in defining ‘influenza-like illness’) [15]
2. tympanic temperature ≥37.5°C (the threshold used in the majority of reports) [12].

Firstly, a Receiver Operating Characteristic (ROC) curve [16] was constructed. ROC curves assess the ability of a test (in this case the ITIS measure) to discriminate between people who have, and who do not have, a condition (fever). The area under the ROC curve for an uninformative test is 0.5.

Secondly, a level of ITIS temperature with sensitivity closest to 85% was chosen and the specificity calculated.

Finally, the positive predictive value (PPV) of the chosen level of ITIS temperature was estimated. The positive predictive value is the proportion of people who test positive (i.e. are ‘positive’ on ITIS) who actually have the condition of interest. It is not appropriate to calculate the PPV of ITIS measures directly in this sample since it was not a random sample of the population of travellers but was instead ‘enriched’ by including as many symptomatic travellers as were prepared to provide respiratory samples. Therefore, the prevalence of fever (by each definition) in the holders of marked questionnaires was combined with the sensitivity and specificity of ITIS for detecting fever to estimate the PPV in the population of all travellers who arrived on the flights that took part in the study. To assess the utility of fever as a screening test for influenza infection (MT-PCR result), sensitivity, specificity, and population PPV for influenza were estimated for each tympanic temperature threshold, and the ITIS threshold used above.

### Ethics

This study was approved by the New Zealand Health and Disability Multiregion Ethics Committee. Written informed consent was obtained from all participants.

## Results

### Participants

**Figure 2:**
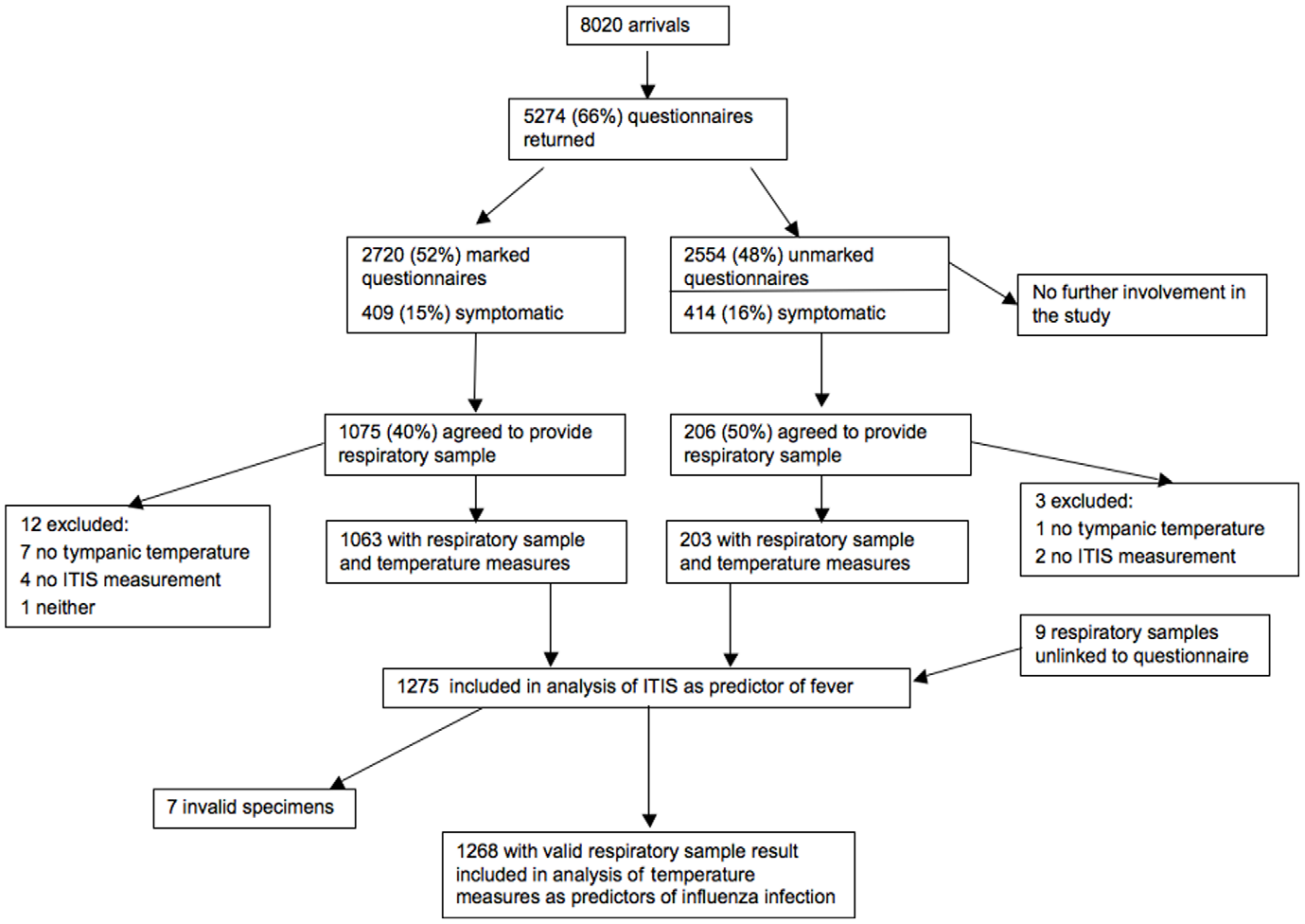
Study flow chart showing how participants were selected from arrivals during the study period.

In total, 5274 travellers returned a questionnaire during the study period, of whom 823 (15.6%) were symptomatic by our definition. Figure 2 shows the pathway of potential participants through the study.

### Accuracy of thermal scanning in predicting core temperature

Seven participants had a tympanic temperature of ≥37.8°C (2 reported no symptoms and 5 were symptomatic). Five held marked questionnaires, giving a prevalence of fever by this definition of 0.5% (5/1063). Half of the 38 participants with a tympanic temperature of ≥37.5°C were symptomatic. Thirty-two of them held a marked questionnaire, so the prevalence of fever by this definition was 3.0% (32/1063).

**Figure 3:**
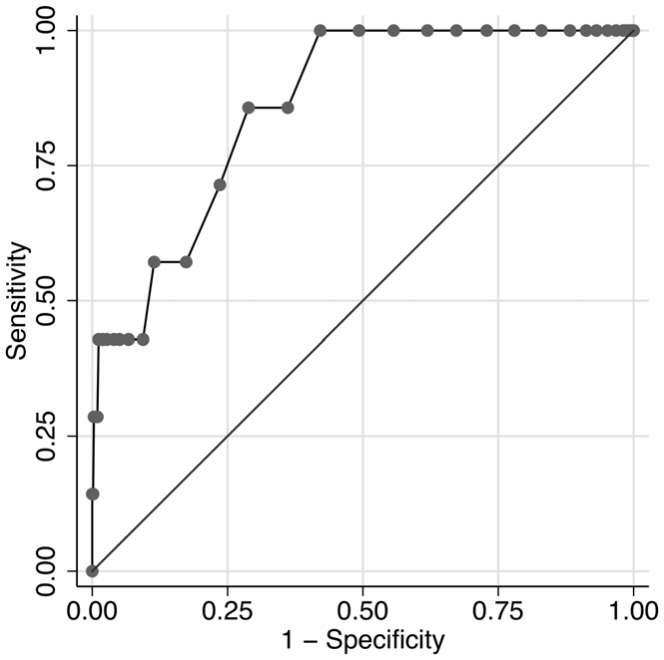
ROC curve of thermal scan vs tympanic temperature ≥37.8°C.

Figure 3 is a ROC curve showing the ability of ITIS front of face measurement to predict a tympanic temperature of ≥37.8°C. Table 1 shows the test characteristics of ITIS as a predictor of tympanic temperature. For each definition of ‘fever’ (determined by tympanic temperature measurement), and for each site of ITIS measurement (front and side of face), the table shows: the ITIS threshold that gave a sensitivity closest to 85% in our data; the proportion of travellers with an ITIS measure above that threshold (i.e. who would have ‘triggered’ the ITIS during screening); the area under the ROC curve; the actual sensitivity of that threshold; and its specificity. The prevalence of fever at each threshold in holders of marked questionnaires (as an estimate of the prevalence in this population of arriving travellers) is also shown, as well as the estimated PPV of ITIS for fever in this population.

**Table 1:** Test characteristics of ITIS as a predictor of tympanic temperature in 1275 arriving travellers.

Definition of fever (tympanic temperature)	ITIS site	ITIS threshold*	Proportion of travellers with ITIS measure above the threshold	Area under ROC curve (95% CI)	Sensitivity	Specificity	Prevalence of fever†	Estimated PPV of ITIS for fever in this population
≥37.8°C	Front of face	35.9°C	29%	0.86 (0.75–0.97)	86%	71%	0.5%	1.5%
	Side of face‡	35.7°C	50%	0.76 (0.54–0.97)	86%	51%		0.9%
≥37.5°C	Front of face	35.4°C	62%	0.71 (0.62–0.81)	84%	39%	3.0%	4.1%
	Side of face‡	35.4°C	69%	0.67 (0.58–0.77)	84%	31%		3.6%

*These were chosen as the threshold with sensitivity closest to 85% in our data.

† in holders of marked questionnaires.

‡ One observation was excluded from these analyses, from an asymptomatic traveller with a tympanic temperature of 37.1 and front of face measure of 37.3 but a side of face measure recorded as 40.1.

### Temperature as a predictor of influenza infection

Of the 1275 respiratory samples obtained from participating travellers, 30 were positive for influenza (3 Type A and 27 Type B), while 7 samples were invalid as they contained no human nucleic acid. The prevalence of influenza infection in holders of marked questionnaires with valid samples was 1.9% (20/1057).

Most (90%; 27/30) influenza-positive participants were symptomatic, but none (0%) had a measured tympanic temperature of ≥37.8°C (99%CI 0% to 18%), and only two (7%) had a measured tympanic temperature of ≥37.5°C(99%CI 0.3% to 31%). Table 2 shows the ability of tympanic and ITIS temperatures to predict influenza infection in a population where the prevalence is 2% (the estimate of the prevalence of infection in this population of arriving travellers). With high sensitivity, specificity is very low. Combined with the low prevalence of influenza infection in this population, PPV is also very low.

**Table 2:** The performance of tympanic and ITIS measures of temperature as predictors of influenza infection in 1268 arriving travellers.

Temperature measure	Area under ROC curve (95%CI)	Temperature threshold*	Sensitivity	Specificity	Estimated PPV for influenza infection (in a population with prevalence = 2%)
Tympanic temperature	0.52 (0.40–0.63)	36.2°C	87%	11%	2.0%
ITIS front of face	0.66 (0.56–0.75)	35.4°C	87%	39%	2.8%
ITIS side of face	0.55 (0.45–0.65)	35.3°C	83%	24%	2.2%

*These were chosen as the threshold with sensitivity closest to 85% in our data.

Influenza-positive participants reported that the first of their symptoms started between 12 hours and 24 days prior to answering the questionnaire, with symptom duration of 2 days or less in 11 participants, more than 2 and up to 5 days in 7 participants, and more than 5 days in 8 participants (3 were asymptomatic and 1 did not respond to this question).

## Discussion

The greatest potential for the use of ITIS to screen incoming or departing travellers for infectious diseases such as a pandemic strain of influenza would be as the first stage of screening; that is, to identify and select out a high risk group for further assessment, for example by questionnaire, body core temperature measurement, and/or respiratory sample collection. This would require very high sensitivity for raised body temperature, as any travellers who ‘slipped through’ the screening process would enter the community and potentially spread infection. In addition, core temperature would need to be a good predictor of infection.

### Can thermal scanning predict core temperature?

This study shows that, among a group comprising both asymptomatic and symptomatic arriving international airline travellers, ITIS can have moderately high sensitivity and specificity for a high body core temperature of ≥37.8°C. However, the low prevalence of fever in arriving travellers means that the PPV is very low.

### Does temperature predict infection?

Measurement of the sensitivity of fever for influenza infection requires that afebrile as well as febrile people, from the same population, are tested for influenza infection. There are few studies that have done this, as symptoms of ‘influenza-like illness’, which include fever, are usually criteria for entry to studies of influenza [17], [18], [19]. Such studies, with selected participants with a high prevalence of influenza infection, overestimate the sensitivity and dramatically overestimate the PPV of fever for influenza infection in unselected populations, such as airline travellers. A review of volunteer challenge studies [20] showed that not only were approximately 30% of influenza infections asymptomatic, but only 35% of those with symptoms had a measured fever >37.8°C. This study found a lower prevalence of fever among the participants infected with Influenza B (7/101) than with Influenza A H1N1(88/285; 31%) [20].

In this study, none of the 30 travellers subsequently identified as infected with influenza (most of whom had influenza B) had a temperature ≥37.8°C, and only two had a temperature ≥37.5°C. A tympanic temperature threshold of 36.2°C would be required to identify a high proportion (87%; table 2) of influenza-infected travellers. The ITIS temperature measures have better specificity than this (non-febrile) level of tympanic temperature for identifying influenza-infected travellers, but PPVs are all low at less than 3%. The ROC result for influenza infection shows that ITIS on their own are not much better than chance at identifying influenza-infected travellers.

These results emphasise what is already known about fever as a symptom of influenza – while it clearly is one of the symptoms that can be experienced by people with influenza infection, it does not occur in all infected people [20]. The prevalence of fever is high in case series of patients with confirmed influenza infection [11], [21], since often one of the criteria that is often used to determine whether testing takes place is the presence of fever. However, where fever is not used as a criterion for influenza testing, the prevalence of fever is by no means 100%, even among people with severe symptoms. For example, among 106 patients hospitalised with respiratory disease [22], 39% of those with confirmed pandemic (H1N1) 2009 infection did not have a temperature of ≥37.8°C at any time during admission. In this study, the predominance of Influenza B infection may partly explain the low prevalence of fever among infected participants (although the three with Influenza A all had tympanic temperatures <37.2°C ).Even with more pyrexigenic strains, among travellers, who by definition are not severely unwell and in fact who are mostly not unwell at all, the proportion of influenza infected people who are afebrile can be expected to be much higher than among hospitalised patients [22] (because the sicker infected people don't travel), as shown in this study.

### Limitations of this study

It was a condition of conducting this study that we did not delay the transit of passengers through the airport by more than a few minutes and, therefore, measurements had to be made efficiently. We used a single measurement by an infrared tympanic thermometer as our ‘gold standard’ measure of core temperature. This approach may have introduced some random error into our results, but is unlikely to have caused systematic bias and is likely to be similar to the way that temperature would be confirmed in practice. In addition, our participants sat still at approximately 1m from the scanner for the ITIS measure and those who were wearing glasses were asked to remove them, steps likely to have provided greater accuracy than ITIS measures that are taken as numerous people walk past a fixed scanner in an arrivals hall. Therefore our study provides an assessment of the best results that could be expected from the use of ITIS in border screening for influenza.

In this study, no influenza-infected travellers had a measured tympanic temperature ≥37.8°C. We do not believe that this was because of systematic errors in tympanic temperature measurements, as these were measured by trained nurses using standard thermometers. We acknowledge that the number of infected travellers was relatively small at 30 but the probability is only 0.005 (0.5%) that the prevalence of fever among the population of infected travellers arriving from Australia into Christchurch at this time was greater than 18%; in other words the vast majority of infected travellers in this population were afebrile.

Among travellers, the proportion of influenza cases who are febrile may be low because those infected with influenza that is causing fever may feel too unwell to travel; 25% of travel-associated cases of pandemic (H1N1) 2009 infection with onset in Singapore were symptomatic on embarkation but the proportion who were febrile was not reported [11]. In addition, it is possible that unwell infected travellers had used anti-pyretics prior to or during the flight, but this is a limitation of ITIS rather than of our study. The study assessed the performance of ITIS in the real world, which includes the fact that some unwell people take anti-pyretics. Also, the flights that were part of this study were relatively short – 3 to 4 hours – and it is possible that on longer flights some of the infected travellers might have become febrile. However, it remains unlikely that fever would occur in all, or even most, infected travellers arriving at any international airport [23].

Good evidence on influenza virus transmissibility during the various phases of viral infection, (including afebrile infection and asymptomatic infection) is not available, but detection of viral RNA on a respiratory sample does not necessarily mean that the infected person is, or will be, infectious. We were not able to perform culture for influenza virus in this study, so it is possible that some of the infected travellers were not shedding viable virus. Although the approximately one third of participants whose symptoms were of 2 days' duration or less were likely to be in the early stages of their infection, those with longer duration of symptoms may not have been. Unfortunately the symptoms of influenza are so non-specific that it is difficult to estimate the stage of influenza infection in a traveller with, for example, a cough that has been present for several weeks. Nonetheless, it seems reasonable to conclude that at least a third, and probably more, of the infected (and afebrile) participants in this study were infectious on or after arrival into New Zealand.

### Implications

Influenza-infected arriving travellers include those who are symptomatic (with or without fever), those who become symptomatic during the flight, those who will develop symptoms following arrival, and those who will never have symptoms. It is not known whether the latter group are infectious, but clearly only the first two categories could potentially be detected by entry screening. Most people who were infected but asymptomatic on boarding will still be asymptomatic on arrival at their destination [23]. However, in the absence of effective exit screening during the H1N1 2009 pandemic, some countries decided to use ITIS in entry screening with the hope that detecting travellers who were febrile on arrival would be worthwhile to reduce the probability of infected travellers entering the country, and that ITIS could detect them [1]. This study provides evidence to the contrary.

The low PPV of ITIS measures for fever in this population means that the number of false positives who would require further investigation, presumably by taking a tympanic temperature, would be very high. In this study, using a front of face ITIS threshold of 35.4°C identifies 69% of travellers as requiring further investigation, of whom only 4.1% had a tympanic temperature ≥37.5°C. The PPV of any of the measures of temperature for influenza infection itself was lower, at less than 3%. However, the prevalence of disease is an important determinant of PPV, and the prevalence of influenza infection in this study, performed during the ‘influenza season’, was low at 1.9%. There are no other published estimates of the prevalence of influenza in arriving travellers, but it could be argued that the prevalence of infection would be higher during a pandemic, which typically infects a higher proportion of the population than seasonal influenza, than in this study. On the other hand, particularly if local containment strategies were in place in originating countries, the prevalence of infection in travellers might be lower during a pandemic. At the beginning of a pandemic, when effective entry screening would be most useful, the prevalence of infection among travellers and therefore the PPV will likely be much lower than the prevalence of seasonal influenza in this study.

More importantly, raised temperature itself by any measurement technology is insufficiently sensitive for influenza infection for its measurement to be effective for mass screening in a pandemic situation. Use of ITIS to identify travellers at high risk of fever, measuring the core temperature of ITIS-positive travellers, and then taking specimens from those with high core temperatures would have failed to identify all the influenza-infected travellers in this study. Using a lower temperature threshold (however measured) for taking specimens could detect a high proportion of influenza-infected travellers only by taking specimens from what is likely to be an unfeasibly high proportion of travellers.

Governments may decide to implement entry screening, including ITIS, for reasons other than to actually detect most influenza-infected arrivals, for example to deter unwell people from travelling, or to demonstrate to their citizens that they are doing everything they can to protect population health. The risks associated with this approach include the potentially very large opportunity cost of further investigating ITIS ‘positive’ travellers, including quarantine of those febrile on tympanic temperature measurement pending specimen processing, and the potential for the loss of public confidence in the pandemic response when it becomes clear that many infected travellers were not detected by the screening and entered the country.

### Conclusion

In this study, during a seasonal epidemic of predominantly influenza type B, influenza-infected arriving travellers had a very low prevalence of fever. Consequently, ITIS would not have identified influenza-infected travellers even though it performed moderately well at detecting febrile travellers. Some aspects of this study may not generalise to a pandemic of Influenza A. Although febrile illness is more common in influenza A infections than influenza B infections, many influenza A infections are afebrile. Our findings therefore suggest that ITIS is unlikely to be effective for entry screening of travellers to detect influenza infection with the intention of preventing entry of the virus into a country.
